# Risikostandardisierte Mortalitätsraten übertreffen als Qualitätsindikator die typischen „Mindestzahlen“ in der onkologischen Chirurgie – ein neuer Blick auf Krankenhauszentralisationen mithilfe nationaler bevölkerungsbezogener Daten

**DOI:** 10.1007/s00066-022-01969-4

**Published:** 2022-07-01

**Authors:** Zsolt Sziklavari, G. G. Grabenbauer

**Affiliations:** 1 Klinik für Thoraxchirurgie, Onkologisches Zentrum Klinikum Coburg, Coburg, Deutschland; 2 Radioonkologie und Strahlentherapie, Onkologisches Zentrum Klinikum Coburg, Coburg, Deutschland; 3 Universitätsklinikum Erlangen, Erlangen, Deutschland

## Hintergrund

Trotz des bekannten Zusammenhangs zwischen jährlichen Patientenzahlen und den chirurgischen Ergebnissen hat sich bislang nur wenig getan, um „Hochrisiko“-Eingriffe nur in „sicheren“ Kliniken durchzuführen bzw. dorthin zu transferieren. Die hier diskutierte Beobachtungsstudie untersuchte, inwieweit das Instrument der „risikostandardisierten“ Letalitätsrate (RSLR) ein besserer Qualitätsindikator für die chirurgische Qualität sein kann als die herkömmliche Patientenzahl pro Einrichtung, das sog. Patientenvolumen.

## Methoden

Aufgenommen wurden 158.079 Patienten, die zwischen 2016 und 2018 an 974 Krankenhäusern in Deutschland mit einem komplexen chirurgischen Eingriff behandelt wurden, und zwar wegen eines Karzinoms der folgenden fünf wichtigsten Diagnosen: Lungen‑, Ösophagus‑, Magen‑, Pankreas- und kolorektales Karzinom. Die Daten von 2010 bis 2015 wurden als Trainingsdatensatz, die von 2016 bis 2018 als Validierungsdatensatz ausgewertet. Für jede chirurgische Gruppe wurden das jährliche Patientenvolumen und die Quintilen der RSLR berechnet und danach diese Grenzwerte auf den Validierungsdatensatz angewendet. Es wurden ein Modell der Ausstiegsstrategie für Kliniken mit schlechten Ergebnissen erarbeitet sowie Effektivität und Effizienz einerseits der volumenbasierten und andererseits der RSLR-basierten Reihung der Kliniken verglichen. Darüber hinaus wurden die Entfernungen oder Fahrtzeiten der Patienten bis zu ihrem nächstgelegenen „Niedrigrisikoklinikum“ für den jeweiligen speziellen Eingriff evaluiert.

## Ergebnisse

Von 2016 bis 2018 wurden 158.079 Patienten in 974 Kliniken operativ behandelt. Mindestens 50 % der sog. „High-volume“-Kliniken waren dabei nicht in der „Niedrigmortalitäts“-Gruppe entsprechend den RSLR-Kriterien enthalten. In einem Zentralisationsmodell entsprechend den RSLR-Daten mussten 32 Patienten in ein „Niedrigrisiko“-Klinikum verlegt werden, um *ein* Menschenleben zu retten, hingegen *47* Patienten in ein „High-volume“-Klinikum. Die mittlere Differenz der Fahrtzeiten zwischen dem nächsten und dem operierenden Klinikum betrug 10 min für die Gruppe der kolorektalen Karzinome und 24 min für die zu operierenden Pankreaskarzinompatienten.

## Schlussfolgerung der Autoren

Risikostandardisierte Mortalitätsraten sind ein vielversprechender Qualitätsindikator zur Einschätzung der chirurgischen Qualität und Letalität. Dieser Indikator übertrifft mit seiner Zuverlässigkeit die häufig genutzte „volumenbasierte“ Betrachtung wegen besserer Effektivität, Effizienz und klinischer Verfügbarkeit.

## Kommentar

Erforderliche Mindestzahlen zur Sicherstellung der Qualität in der onkologischen Chirurgie: Da stehen Ösophagus- und Magenkarzinome ebenso wie Eingriffe bei Lungen‑, Pankreas- und Rektumkarzinomen im Zentrum des Interesses. Sie werden hierzulande sowohl von den Krankenkassen als auch von den Zertifizierungsinstitutionen der Deutschen Krebsgesellschaft seit Jahren wie ein heiliger Gral abgefragt. Und mehr noch: Gefühlt werden alle 3 bis 5 Jahre diese Zahlen auf der Basis „evidenzbasierter Daten“ um 30–50 % erhöht. Wozu führt das bzw. könnte das führen? Einmal besteht die problematische Option für kleinere Häuser, die aufwendige onkologische Chirurgie an „Niedrigrisiko“-Kliniken, also an größere Leistungserbringer abzutreten. An mittleren Häusern, die möglicherweise die geforderten Mindestzahlen nicht bzw. oft nur knapp erreichen, besteht die nicht zu unterschätzende Gefahr, dass Indikationen für onkologische Eingriffe an „Problempatienten“ gestellt werden, die wegen erheblicher operativer Risiken dafür nicht geeignet sind. Insofern erscheint es uns verdienstvoll, dass sich die Kollegen der Heidelberger Thoraxklinik der Frage „Qualität vs. Quantität“ gewidmet haben. Sie zeigen, dass „risikostandardisierte Letalitätsraten“ (RSLR) ein besserer Qualitätsindikator sind als die typische „volumenbasierte“ Betrachtung. Interessant ist dabei die praktische Konsequenz: Wie viele Patienten müssen z. B. in ein Klinikum mit niedrigerer Letalität verlegt werden, um ein einziges Leben zu retten? Hier wird deutlich, dass das RSLR-basierte Modell mit einer niedrigeren Verlegungsquote auskommt als die volumenbasierte Vorgehensweise. Diese ist beim Magenkarzinom um 20 % geringer, beim Ösophagus‑, Lungen- und Pankreaskarzinom um 30 % und um 40 % beim Kolon- und Rektumkarzinom. Dies spricht eindeutig für die Notwendigkeit, weiterhin innovative Strategien zu entwickeln, mit denen wir die Qualität in der chirurgischen Onkologie definieren und verbessern können [1]. Gleiches gilt sicher auch für andere Verfahren in der Onkologie.

Dennoch weist diese Beobachtungsstudie auch eine Reihe von Schwachstellen auf, die ihre Aussagekraft limitieren: Erstens hatten die Kollegen in Heidelberg keine Informationen über die Patientenselektionen erhalten, auch nicht über die TNM-Gruppierung und die Tumorhistologie. Zweitens verfolgten die Autoren nach der Entlassung der Patienten deren Todesraten nicht weiter. Darüber hinaus müssten die publizierten Ergebnisse international validiert werden, insbesondere in Anbetracht der verschiedenen nationalen Gesundheitssysteme mit großen Unterschieden in der geografischen Zuordnung des Patientenwohnorts bzw. in den Entfernungen zum ausgewählten aktiven Krankenhaus.

Verbündete auf dem Weg zur Verbesserung der Qualitätsstandards in der Onkologie sind verständlicherweise die Zertifizierungskommissionen, welche verpflichtend die Weiterentwicklung und Sicherung der Qualitätsstandards in der Medizin unterstützen. So beauftragte der Gemeinsame Bundesausschuss (G-BA) 2018 das Institut für Qualität und Wirtschaftlichkeit im Gesundheitswesen (IQWiG) mit einer systematischen Literaturrecherche zur Evidenzbewertung des Zusammenhangs zwischen der Leistungsmenge und den onkologischen Resultaten [2]. Der hier diskutierte Report dient dabei aktuell als Grundlage für die Einführung der Mindestmengenregelung in der Thoraxchirurgie. Zu beachten ist, dass die Mehrheit der Beobachtungsstudien (22 von 23) nicht in Deutschland durchgeführt wurden. So war es unumgänglich, die vergleichende Aussagekraft aller 23 eingeschlossenen Studien im Hinblick auf deren Ergebnisse (Gesamtletalität, therapieassoziierte Letalität, 30- und 90-Tage-Letalität, Krankenhausletalität und Morbidität bzw. Wiedereinweisung) zu betrachten; sie wurden mit einem niedrigen Risiko bewertet.

Für die Zielgröße Gesamtüberleben und therapieassoziierte Letalität – bei niedriger Aussagekraft der Ergebnisse – zeigte sich ein positiver Zusammenhang zwischen der Leistungsmenge pro Krankenhaus und der Qualität der Behandlungsergebnisse. Man sah aber keinen direkten Zusammenhang zwischen der Leistungsmenge und der Qualität des Behandlungsergebnisses hinsichtlich der Zielgrößen 30- und 90-Tage-Letalität. Studienübergreifend zeigte sich für die Zielgröße „Versterben im Krankenhaus“ bei niedriger Aussagekraft der Ergebnisse ein überwiegend positiver Zusammenhang zwischen der Anzahl von Lungenresektionen pro Krankenhaus und der Qualität des Behandlungsergebnisses, wobei die Resultate für die verschiedenen Resektionsverfahren heterogen waren. Somit bleiben aus unserer Sicht Quantität und auch die risikostandardisierte Letalitätsrate nur Mittel zum Zweck. Quantität und Erfahrung sind mit Sicherheit wichtig, führten in verschiedenen Publikationen aber nicht automatisch zu höherer Qualität [3–5].

## Fazit

Anstatt eine strikte Grenze im Sinne eines vorgeschriebenen Patientenvolumens pro Institution und Eingriff zu setzen, sollten wir interdisziplinär eher nach qualitätsbasierten und damit innovativen Wegen suchen, wie Kliniken mit mittlerem und hohem Volumen weiterhin schnell, sicher und innovativ Tumorpatienten vor Ort behandeln können.

*Zsolt Sziklavari und Gerhard G. Grabenbauer, Coburg*
